# NIA Long Life Family Study: Objectives, Design, and Heritability of Cross-Sectional and Longitudinal Phenotypes

**DOI:** 10.1093/gerona/glab333

**Published:** 2021-11-05

**Authors:** Mary K Wojczynski, Shiow Jiuan Lin, Paola Sebastiani, Thomas T Perls, Joseph Lee, Alexander Kulminski, Anne Newman, Joe M Zmuda, Kaare Christensen, Michael A Province

**Affiliations:** 1 Division of Statistical Genomics, Department of Genetics, Washington University School of Medicine, St Louis, Missouri, USA; 2 Biostatistics, Epidemiology, and Research Design Center, Institute for Clinical Research and Health Policy Studies, Tufts University, Boston, Massachusetts, USA; 3 Department of Medicine, Geriatrics Section, Boston Medical Center, Boston University School of Medicine, Boston, Massachusetts, USA; 4 Taub Institute for Research on Alzheimer’s Disease and the Aging Brain, Department of Neurology, Columbia University Medical Center, New York, New York, USA; 5 Biodemography of Aging Research Unit, Social Science Research Institute, Duke University, Durham, North Carolina, USA; 6 Department of Epidemiology, Graduate School of Public Health, University of Pittsburgh, Pittsburgh, Pennsylvania, USA; 7 Department of Human Genetics, Graduate School of Public Health, University of Pittsburgh, Pittsburgh, Pennsylvania, USA; 8 Unit of Epidemiology, Biostatistics and Biodemography, Department of Public Health, Southern Denmark University, Odense, Denmark

**Keywords:** Growth curves, Healthy aging, Heritability, Longevity, Longitudinal change

## Abstract

The NIA Long Life Family Study (LLFS) is a longitudinal, multicenter, multinational, population-based multigenerational family study of the genetic and nongenetic determinants of exceptional longevity and healthy aging. The Visit 1 in-person evaluation (2006–2009) recruited 4 953 individuals from 539 two-generation families, selected from the upper 1% tail of the Family Longevity Selection Score (FLoSS, which quantifies the degree of familial clustering of longevity). Demographic, anthropometric, cognitive, activities of daily living, ankle-brachial index, blood pressure, physical performance, and pulmonary function, along with serum, plasma, lymphocytes, red cells, and DNA, were collected. A Genome Wide Association Scan (GWAS) (Ilumina Omni 2.5M chip) followed by imputation was conducted. Visit 2 (2014–2017) repeated all Visit 1 protocols and added carotid ultrasonography of atherosclerotic plaque and wall thickness, additional cognitive testing, and perceived fatigability. On average, LLFS families show healthier aging profiles than reference populations, such as the Framingham Heart Study, at all age/sex groups, for many critical healthy aging phenotypes. However, participants are not uniformly protected. There is considerable heterogeneity among the pedigrees, with some showing exceptional cognition, others showing exceptional grip strength, others exceptional pulmonary function, etc. with little overlap in these families. There is strong heritability for key healthy aging phenotypes, both cross-sectionally and longitudinally, suggesting that at least some of this protection may be genetic. Little of the variance in these heritable phenotypes is explained by the common genome (GWAS + Imputation), which may indicate that rare protective variants for specific phenotypes may be running in selected families.

Longevity is associated with compression of disability (1,2) at extreme ages, morbidity is compressed as well (3). The familiality of such compression and underlying causative factors are unknown. Individuals differ markedly in rates of age-related changes in differing functions that influence the risk of morbidities in late life. The National Institute on Aging Long Life Family Study (LLFS) was designed to assess genetic and behavioral/environmental risk factors associated with exceptional longevity by employing a family-based cohort design. The exceptionally healthy LLFS population provides a unique resource to identify familial and genetic factors contributing to favorable cross-sectional profiles and trajectories of change with age. LLFS is a unique multicenter, multinational resource for discovering protective alleles and associated biological signatures for Healthy Aging Phenotypes (HAPs) and Exceptional Longevity (EL) (4,5). No other study has LLFS’ size and degree of exceptional familial longevity, as fewer than 1% of Framingham Heart Study (FHS) families would meet the EL selection criteria for LLFS (6). Furthermore, specific HAPs may constitute differing pathways or mechanisms to exceptional longevity, which is exhibited among LLFS families. Some LLFS families demonstrate longer health spans and marked delays in the onset of, for example, dementia and heart disease (3,7,8), whereas other families demonstrate healthy metabolic profiles (9). Furthermore, there is a limited overlap of families that demonstrate clustering of individuals with exceptional memory, grip strength, pulmonary function, blood pressure, and/or metabolism (8,10–13) Additionally, the delay in many of these diseases is observed not only in the LLFS older generation but also in the offspring generation, who are in their 60s through early 80s (14). Here we describe the study design, family selection criteria, demonstration of favorable phenotypes on average, heterogeneity of families and calculation of change trajectories for key healthy aging phenotypes, and their heritabilities.

## Method

### Identification of Families

The U.S. field centers used Center for Medicare and Medicaid Services lists of Medicare enrollees to mail a recruitment brochure. The initial file included people who were at least 79 years old on January 1, 2005; had no recorded date of death; were not in the end-stage renal disease or hospice programs; lived in zip codes near (within 3 hours driving distance) one of the 3 U.S. study centers (Boston University Medical Center in Boston, MA, Columbia College of Physicians and Surgeons in New York, NY, and the University of Pittsburgh in Pittsburgh, PA). An age/sex stratified pilot mailing tested the yield of exceptionally long-lived families recruited from mailing to individuals in their 80s versus higher age strata (*N* = 881 responses). Based on these yields, subsequent mailings targeted those age 89 and older. Study participants were also recruited from local communities using mailed brochures, posters, web-based media and newspaper advertisements, as well as community-based presentations. Additional mailing lists were obtained through voter registries and purchased public domain lists from various commercial vendors (8).

The University of Southern Denmark field center identified individuals who would be ages 90 and above during the study recruitment period through the Danish National Register of Persons, which contains current information on names, including past names such as maiden names for women, addresses, place of birth, marriages, and vital status (15). Archived parish registers in Denmark were searched for information on the place of birth and the names were searched to locate the parents of the older adults to identify sibships. Based on the above information, 659 potentially eligible families were identified ranked by the Family Longevity Selection Score (FLoSS). Contact was made with potential probands to further assess the family’s eligibility for and willingness to participate in the LLFS using criteria parallel to that used in the United States.

### Family Longevity Selection Score

The FLoSS was developed to quantify the degree of familial longevity for inclusion in the LLFS (6). There are 2 components to the score. The first component is based upon the (log) tail probability of each sibling’s survival status (living or dead) derived from the appropriate age/sex/birth cohort life tables. The values are summed to obtain a score for the entire sibship. A value of zero in this component indicates survival exactly as expected from the life tables, and the higher the values the more the survival experience of that sibship exceeds that expected for the population. The score is scaled so that the expected value is independent of sibship size. The second component adds a “bonus” for each living sib since we wanted to identify larger families for prospective evaluation. To be eligible, a proband sibship had to have a FLoSS of 7 or greater and a minimum family size of 3 (specifically the proband, at least one living sibling, and one offspring), all willing and able to give informed consent and participate in the interview and in-home examination including providing a blood sample for serum and DNA extraction. Based on our CMS pilot data, as well as comparison to the FHS families, we determined that using these selection criteria represents the upper < 1% tail of the exceptional survival distribution. Thus, the least exceptional family in LLFS shows more extreme familial longevity than 99% of the Framingham Heart Study families.

### Establishment of the LLFS Cohort: Visit 1

The original recruitment and examination for LLFS were conducted over a 3-year period, April 2006–May 2009. LLFS successfully enrolled and extensively phenotyped 4,953 individuals from 539 two-generational families that demonstrated clustering for exception longevity in the upper generation (ie, with a sibship FLoSS > 7) from 3 US field sites (Boston University, Boston, MA; Columbia University, New York, NY; and University of Pittsburgh, Pittsburgh, PA) and one field site in Denmark (University of Southern Denmark, Odense, Denmark; Table 1). These families included long-lived individuals (generally 90+), their siblings, spouses, offspring of all siblings, and spouses of the offspring (to serve as an internal control group). The average family size was 9.1 members (range 3–79). 55.2% of the cohort at the time of enrollment was female, and 99% was white. The mean age of the proband generation was 90.2+6.6 years (range 55–110), and that of the offspring generation was 61.2+8.4 years (range 25–88). The average FLoSS was 11.7 (range 7–33.9).

**Table 1.** Characteristics of the LLFS Sample by Generation and Field Center

		Boston U		U Pittsburgh		Columbia U		Denmark		Overall	
Visit 1: 2006–2009											
Families		134		159		170		76		539	
Generation		Proband	Offspring	Proband	Offspring	Proband	Offspring	Proband	Offspring	Proband	Offspring
*N*		456	852	488	816	523	555	260	1 003	1 727	3 226
Age ± *SD*		90.2 ± 7.3	61.0 ± 8.1	89.7 ± 6.1	60.6 ± 8.5	90.0 ± 6.5	61.0 ± 8.4	91.2 ± 6.3	61.9 ± 8.5	90.2 ± 6.6	61.2 ± 8.4
Range		55–110	33–88	72–105	37–87	67–109	25–88	w65–104	36–88	55–110	25–88
% Female		57.7	55.5	51.8	57.6	54.2	56.3	63.1	52.2	55.7	55.2
% White		99.2	99.3	99.4	99.7	97.4	98.1	100.0	99.9	98.9	99.4
Family size	Mean	9.5		8.2		6.4		16.7		9.1	
	Range	3–49		3–43		3–21		3–79		3–79	
FLoSS	Mean	12.1		10.8		11.2		14.8		11.7	
	Range	7.0–33.9		7.0–29.8		7.0–29.8		8.0–28.0		7.0–33.9	
Visit 2: 2014–2017											
Generation		Proband	Offspring	Proband	Offspring	Proband	Offspring	Proband	Offspring	Proband	Offspring
*N*		95	651	147	541	174	466	62	768	478	2 426
Age ± *SD*		91.4 ± 8.2	67.6 ± 7.7	93.5 ± 6.1	68.0 ± 8.0	93.6 ± 6.4	68.1 ± 7.9	92.7 ± 6.8	69.4 ± 7.7	93.0 ± 6.8	68.4 ± 7.9
Range		56–107	40–95	79–106	45–93	65–108	42–89	72–110	46–95	56–110	40–95
% Female		61.1	53.3	61.2	56.6	55.1	56.9	72.6	52.9	61.5	54.6
% White		97.9	99.9	99.3	99.6	97.7	98.1	100.0	99.2	97.9	99.3

*Note:* FLoSS = Family Longevity Selection Score; LLFS = Long Life Family Study.

### Components of In-Home Exams

Interviews and examinations were conducted in the home setting with portable equipment by centrally trained and certified research assistants using a common protocol. All family members, regardless of age, had the same assessment battery. At the baseline examination, all participants had to be able to give informed consent to participate. At follow-up, participants who were ill or who had incident dementia continued to participate whether proxy consent was obtained and they could express assent and cooperation. We used proxy interviews for selected questionnaires where participants were unable to answer for themselves due to illness. If an in-home visit was not feasible, we conducted a comprehensive telephone interview and obtained biological specimens (blood or saliva sample) using an outside laboratory or physician’s office. Selected measures were designed to assess aspects of EL that (i) have significant heritability, (ii) are related to longevity and healthy aging, and (iii) can be assessed in the home setting. In Table 2, we give more details about the items obtained within each healthy aging phenotype domain and give a comparison to what is measured in FHS. In-home visits last an average of 3 hours per participant and included cognitive, physical, spirometry, and carotid artery ultrasound scans (year 2 only). Blood specimens were collected at home visits and banked at the Central Biospecimen Laboratory at the University of Minnesota and included sera, whole blood, DNA extraction, PAXgene tubes for RNA profiling, and banked lymphocytes.

**Table 2.** LLFS Exceptional Survival Phenotypes and Environmental Exposure Measures in Visit 1, Annual Follow-up, and Visit 2

LLFS Core Phenotypes	Interview; Physical Exam; Biospecimen Repository Performed in Visit 1	Annual Phone Follow-up. “Expanded” f/up Performed Each Year for G1, Every 3 y for G2 < 70 y old and Annually for G2 ≥ 70 y	Visit 2	Measured in Framingham Heart Study
Age	Validated age or age at death, Family history of longevity longevity	Update vital status (annual and expanded)	Update vital status	Age of death
Disability-free survival				
Cognitive function	Medical history; Clinical Dementia Rating Scale, MMSE, Logical Memory—Immediate, Digit Span Forward, Digit Span Backward, Category Fluency, Digit Symbol Substitution Test, Logical Memory—Delayed	Telephone Interview for Cognitive Status (TICS) and Dementia Questionnaire (DQ) (expanded)	Same as visit 1 but adds digital pen and digital voice data, HVLT, Clock drawing, Trail Making, Letter fluency	MMSE Neuropsych arm: category fluency, clock drawing, trail making, letter fluency
Physical function	ADLs; grip strength, gait speed, balance, chair stands, heart rate	IADLs, ADLs (annual and expanded)	Same as visit 1, IADL, and Pittsburgh Fatigue Scale	Grip, gait speed
Disease-free survival				
CVD	Medical history; blood pressure (BP), ankle-brachial index (ABI)	Medical history update (expanded)	Visit 1 + carotid ultra sound	Medical history, BP, ABI, carotid
Cancer	Medical history	Update (expanded)	Update	Similar
Lung disease	Medical history; FEV_1_, FEV_6_ with portable spirometer	Medical history update (expanded)	Update	Medical history, FEV_1_
Diabetes	Medical history, medication use; fasting glucose and insulin, weight, waist circumference, height, knee height	Medical history, medication use update (expanded)	Update (no insulin)	Medical history, weight, height, waist
Renal disease	Medical history; see labs below	Update (expanded)	Update	Medical history, creatinine
Depression/personality	CES-D, neuroticism, extraversion, openness (NEO) 2 factors only	Full 5-factor NEO (expanded once)	CES-D	
Environmental/behavioral exposures				
Social	Place of birth, education	Not needed	No	Similar
Habits	Smoking, alcohol consumption, physical activity (current and historical)	Physical activity and sleep habits (one time follow-up)	Update	Similar
Health care	Utilization, classes of medications	Update (annual and expanded)	Update	Similar
Nutrition	Weight history	Not collected	Update	Similar
Reproduction	Parity, age of last pregnancy, age at menopause, hormone replacement therapy	Medication update (expanded)	Update if age < 65	Age at menopause
Laboratory studies				
Genetics	Leukocytes or buccal cells for DNA, future lymphoblastoid cell lines. Telomere studies	None	Repeated	Genome-wide genotype data
Other	Fasting glucose, insulin, HbA1C, creatinine, cystatin C, total/HDL/LDL cholesterol, hemoglobin, leukocyte, and platelet counts. Iron, TIBC, ferritin, IL6, heat shock protein 60 and 70. 10 aliquots serum + plasma for future analysis	None	*	Fasting glucose, insulin, lipids

*Notes:* ADL = activities of daily living; CES-D = Center for Epidemiological Studies Depression Scale; CVD = cardiovascular disease; LLFS = Long Life Family Study; MMSE = Mini-Mental State Examination.*Due to budget limitations, limited biochemical analyses were performed on visit 2 specimens. In the current funding period, we are repeating visit 1 assays on stored visit 2 biospecimens.

### Annual Follow-up

Annual telephone follow-up is conducted to update vital status and medical conditions. Expanded follow-up is conducted every year for the proband generation and includes reported physical function, activities of daily living, and telephone assessed-cognitive function. Because they are generally so much younger these items are assessed only every 3 years for the offspring generation. However, once an offspring generation participant reaches age 70, they are given the expanded follow-up yearly. Annual follow-up has a high completion rate of 81%–85%, depending on the generation. To encourage continued engagement in the study, the Field Centers send holiday greeting cards, calendars, birthday cards, and short research updates as well as an annual newsletter.

### Mortality of the Cohort and Valid Cause of Death

Seventy-three percentage of the proband generation and 7% of the offspring generation have died according to family members and regular queries to the National Death Index. We performed a cause of death adjudication for all reported deaths in the United States (for the Denmark Field Center, validated causes of death are obtained through the centralized Danish Medical System). An Adjudication Committee consisting of 4 physicians evaluates all relevant collected information on each reported death, to determine immediate, primary, and contributing causes of death, following a standard protocol. Records evaluated include death certificates, hospital, nursing home, and medical records, as well as next of kin reports. This has been particularly important for identifying dementia-related deaths, which remain underreported on death certificates.

### Genotyping and Sequencing

Genome-Wide Association data were generated using Illumina 2.5M Omni SNP array, which was imputed to 38M SNPs using 1000 Genomes imputation, resulting in a number of publications (16–21) and which have been shared with the broader scientific community via dbGaP (dbGaP Study Accession: phs000397.v1.p1). We also performed targeted exome sequencing of selected candidate genes for rare coding variation associated with health and longevity in all participants (22). An LLFS ancillary pilot study conducted Whole Exome Sequencing on one subject per pedigree. Approximately 40 circulating biomarkers that include (but not limited to) lipid metabolism, hematologic profiles, an array of endocrine biomarkers, kidney function, inflammation, and glucose homeostasis were measured in all participants. The age and sex distributions of these age-related biomarkers have been reported (23).

### Longitudinal Change: Visit 2

A second in-home evaluation (Visit 2) was conducted on LLFS participants approximately 8–10 years after Visit 1, from 2014 to 2017 (Table 1). We repeated measures that were expected to show change over time, updated medical history and medications, and repeated a blood draw. We enhanced the Visit 2 examination by adding in-home portable carotid ultrasound to better define vascular health (Table 2).

### Estimation of Personal Longitudinal Change Phenotypes: Random Coefficient Models (a.k.a. Growth Curves)

Longitudinal studies can characterize rates-of-change in aging-related phenotypes, both at a group level and at a personal level. For a study such as LLFS, where we have evidence that there is overall protection, but at the same time considerable heterogeneity, it is important to accurately assess *individual longitudinal change* for each LLFS participant for every key phenotype. The most obvious way to calculate individual change slope is to simply calculate the change in phenotype per unit time (ie, the difference between the participant’s Visit 2 and Visit 1 Phenotype, divided by the time between the visits). This is equivalent to fitting a separate Ordinary Least Squares (OLS) regression model individually to each participant. However, if phenotypes are measured with error (as they always are to at least some extent), then these measurement errors accumulate in these calculations—they do not cancel. In fact, for independent measurement errors, the error variance of the change in phenotype between the 2 visits is the sum of the 2 visit error variances. The increase in error in calculating individual change with this simple approach reduces the ability to assess the impact of risk and/or protective factors influencing individual trajectories. In fact, as demonstrated in the following section, the heritabilities of these simple personal OLS estimates of change are virtually zero because the increase in error variance overwhelms any heritability signal. Consequently, we estimated individual longitudinal change using a random coefficient model (RCM), also known as growth curve model (24). This approach has been particularly useful in estimating the longitudinal change in genetic and family studies, including LLFS (eg, (25–28)). The RCM properly models the error in phenotype measurement at each time point and thereby avoids the increase in error variance which is the root problem of the simpler individual OLS approach. The mathematical details of the RCM are given in Supplementary Appendix 1, but in concept, the model simultaneously estimates each participant’s personal trajectory while also using all participant’s data by making the additional assumption that the population of intercepts and slopes are multivariately normally distributed with an unknown variance–covariance matrix. This produces smoothed individual slopes and intercepts, trading bias for increased precision. The RCM effectively transforms the individual data from outcomes at each time point to individual slopes and intercepts for each participant. These individual slopes and intercepts then become new phenotypes for evaluation, and in particular, the RCM slopes are estimates of individual trajectories with age. We systematically generated individual growth curve trajectories for all key phenotypes and all participants in LLFS, and calculated the heritabilities of each cross-sectionally and longitudinally (Table 3).

**Table 3.** LLFS Phenotypes: Cross-Sectional and Longitudinal Heritabilities

Phenotype	Cross-Sectional		Longitudinal		
	P1 = Phenotype @ Visit 1 Heritability	P2 = Phenotype @ Visit 2 Heritability	Growth Curve		Δ = (P2 − P1)/(T2 − T1) OLS Slope Heritability
			Corr. Between Measured Pi & GC Est.	GC Slope Heritability	
Cardiovascular traits					
Systolic blood pressure	0.23	0.19	0.87	0.30	0.00
Diastolic blood pressure	0.23	0.18	0.87	0.26	0.08
Pulse pressure	0.23	0.26	0.86	0.37	0.00
Heart rate	0.34	0.33	0.88	0.52	0.00
Ankle–brachial index*	N/A	0.16	NA		NA
Carotid IMT	N/A	0.47	N/A		N/A
Far wall carotid IMT	N/A	0.37	NA		NA
Lumen diameter, CCA	N/A	0.53	N/A		N/A
Adventitial diameter, CCA	N/A	0.64	N/A		N/A
Plaque burden index	N/A	0.30	N/A		N/A
Anthropometric traits					
Body mass index	0.47	0.55	0.97	0.56	0.13
Weight	0.57	0.61	0.97	0.64	0.13
Abdominal circumference	0.45	0.57	0.95	0.56	0.18
Arm span*	N/A	0.74	N/A		N/A
Lipid traits					
Cholesterol (total)	0.36	0.40	0.89	0.44	0.06
HDL	0.48	0.52	0.94	0.50	0.05
LDL	0.44	0.43	0.91	0.50	0.04
Triglycerides	0.34	0.29	0.90	0.36	0.00
Glycemic traits					
Glucose	0.33	0.26	0.84	0.39	0.23
Hemoglobin A1c	0.42	0.40	0.91	0.42	0.17
Insulin	0.27	N/A	N/A		N/A
Lung-related traits					
FEV_1_ (1 s)	0.46	0.40	0.97	0.12	0.04
FEV_6_ (6 s)	0.48	0.44	0.96	0.14	0.07
Forced vital capacity (FVC)	0.46	0.43	0.96	0.15	0.08
FEV_1_/FVC	0.32	0.39	0.96	0.02	0.00
%Predicted FEV_1_ (30–150)	0.31	0.29	0.90	0.32	0.04
%Predicted FEV_6_ (30–150)	0.26	0.28	0.85	0.28	0.07
Blood-based biomarkers					
Telomere length	0.53	N/A	N/A		N/A
Creatinine	0.32	0.26	0.90	0.35	0.15
Cystatin C	0.33	N/A	N/A		N/A
Interleukin-6	0.21	N/A	N/A		N/A
C-reactive protein (hsCRP)	0.24	N/A	N/A		N/A
ProBrain Natriuretic Peptide, N-Terminal (pro-BNP)	0.22	N/A	N/A		N/A
Vitamin D2	0.16	N/A	N/A		N/A
Vitamin D3	0.30	N/A	N/A		N/A
Transferrin	0.41	N/A	N/A		N/A
sRAGE	0.38	N/A	N/A		N/A
Testosterone	0.13	N/A	N/A		N/A
Insulin-like growth factor (IGF-1)	0.38	N/A	N/A		N/A
DHEA sulfate	0.39	N/A	N/A		N/A
Albumin	0.34	N/A	N/A		N/A
Physical performance measures					
Grip strength (kg)	0.44	0.47	0.89	0.52	0.29
Gait speed (m/s)	0.23	0.15	0.92	0.29	0.09
Chair stand	0.22	0.25	0.85	0.36	0.00
Physical perform (SPPB)	0.14	0.08	0.82	0.12	0.00
Framingham physical activity score	N/A	0.17	N/A		N/A
Pittsburgh fatigability score					
Mental component	N/A	0.24	N/A		N/A
Physical component	N/A	0.22	N/A		N/A
Neuropsychological traits					
MMSE	0.19	0.22	0.80	0.08	0.04
CES-D	0.15	0.15	0.93	0.15	0.07
Logical memory, immediate	0.28	0.28	0.87	0.39	0.01
Trail Making Part A	N/A	0.31	N/A		N/A
Trail Making Part B		0.32			
Hopkins Verbal Learning Test (HVLT)	N/A	0.33	N/A		N/A
Digit Span Forward	0.35	0.46	0.88	0.53	0.13
Digit Span Backward	0.30	0.29	0.86	0.40	0.05
Category Fluency—Animal	0.36	0.35	0.88	0.48	0.03
Digit Substitution Test	0.40	0.45	0.94	0.54	0.03
Logical memory, delayed	0.28	0.25	0.87		0.38
Healthy Aging Index (HAI)					
Evenly weighted HAI	0.28	0.27	0.88	0.30	0.11
Mortality weighted HAI	0.26	0.27	0.89	0.27	0.16

*Notes:* CCA = Common Carotid Artery; CES-D = Center for Epidemiological Studies Depression Scale; DHEA = dehydroepiandrosterone; FEV_1_ = forced expiratory volume in 1 s; FEV_6_ = forced expiratory volume in 6 s; HDL = high-density lipoprotein; IMT = intima-media thickness; LDL = low-density lipoprotein; LLFS = Long Life Family Study; MMSE = Mini-Mental State Examination; N/A = not applicable; SPPB = Short Physical Performance Battery. “P1=Phenotype @ Visit 1 Heritability” is the heritability of the phenotype measured at Visit 1. “P2=Phenotype @ Visit 2 Heritability” is the heritability of the phenotype measured at Visit 2. “Corr. between Measured Pi & GC Est.” is the Pearson correlation between the phenotype as measured (in either Visit 1 or Visit 2) and the estimated phenotype at that visit by the growth curve model. “GC Slope Heritability” is the heritability of the growth curve individual slopes (personal rate of change per unit time). “Δ = (P2 − P1)/(T2 − T1) OLS slope Heritability” is the heritability of the individual Ordinary Least Squares Slopes, which for two visits is the simple difference between Visit 2 and Visit 1 phenotype divided by the time between visits.

### Estimation of Heritability

#### Exclusions and covariate adjustment

Exclusions were made based on each phenotype as follows. For systolic blood pressure (SBP), diastolic blood pressure (DBP), pulse pressure, and pulse rate, modifications to the data were made if participants were taking antihypertensive medications (SBP+15 and DBP+10). For glucose and hemoglobin A1c, analyses were only performed on nondiabetics. Participants were defined as having diabetes if their fasting glucose was ≥126 mg/dL or their glycated hemoglobin was ≥6.5 or they reported current diabetes or were taking diabetes medications. Participant values were set to missing if they reported diabetes or were taking medications for diabetes; glucose values were also set to missing if the participant was nonfasting. For lipid traits, values were set to missing if the participants were taking lipid-lowering medications or were not fasting.

Visit 1 values were adjusted for gender, age at Visit 1, age at Visit 1 squared, and field site. Visit 2 values were adjusted for gender, age at Visit 2, age at Visit 2 squared, and field site. The change from Visit 1 to Visit 2, Δ = ((Visit 2−Visit 1)/years passed), was adjusted for gender, baseline age, baseline age squared, and field site. Years passed is calculated as (date of Visit 2 − date of Visit 1)/365.25. The individual growth curve slope phenotypes were adjusted for gender, baseline age, baseline age squared, and field site.

The basic heritability analyses of selected key LLFS phenotypes, summarized in Table 3, were calculated with 2 major assumptions: (i) ignore the potential impact of mortality selection on heritability and (ii) restrict the calculation to those participants who have both Visit 1 and Visit 2 measurements for each phenotype (ie, estimating conditional heritabilities). Heritability estimation for the longitudinal slopes was performed using Sequential Oligogenic Linkage Analysis Routines (SOLAR) by employing a pedigree-/kinship-based model.

## Results

**Figure 1.**
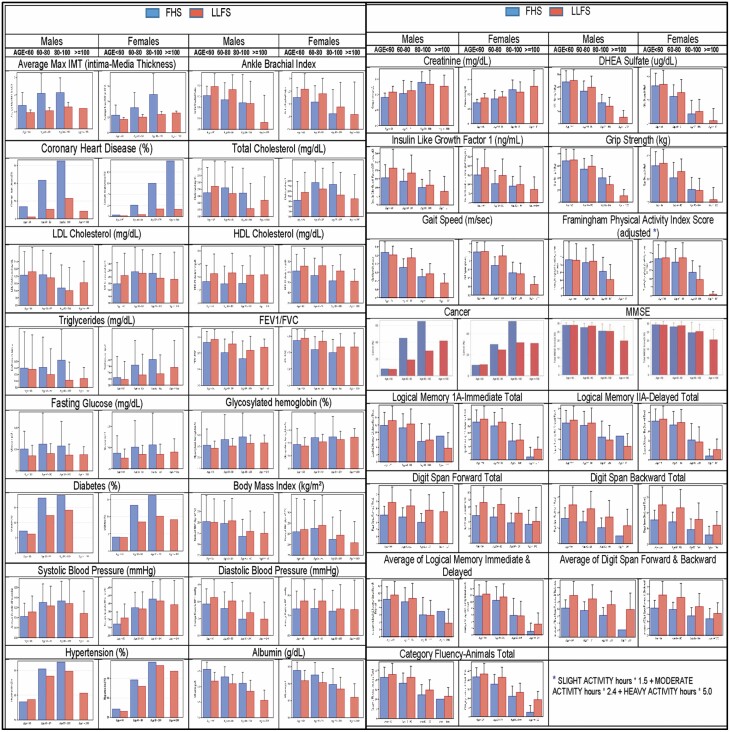
Comparison of key phenotypes between LLFS and FHS in age/sex groups. Bar heights indicate mean with standard error whiskers comparing LLFS with FHS in sex/age groups. FHS = Framingham Heart Study (blue). LLFS = Long Life Family Study (red).

Previously published findings have demonstrated that the LLFS families have lower rates of many of the diseases of older adults, and show healthier aging profiles than other random sample cohorts (5,8,29–33). In Figure 1, we show a comparison of key phenotypes between the LLFS and an age–sex-matched sample from FHS, in age categories of <60, 60–80, 80–100, and >100, using cross-sectional data (first measurement on each subject for each measure). We considered all measures where we wanted to harmonize the LLFS measures with the Framingham study. Note that across age/sex groups, LLFS participants have a lower average maximum intima-media thickness (IMT; ie, lower atherosclerotic burden), lower rates of coronary heart disease, healthier HDL cholesterol levels and pulmonary function (FEV_1_/FVC), and lower age/sex prevalence of diabetes and hypertension. These findings are all the more remarkable, considering that BMI is one of the few risk factors that does NOT show a lower profile in LLFS. In fact, the age-/sex-specific prevalence of obesity is nearly identical for the 2 studies. With regard to cognitive functioning, the LLFS cohort shows higher performance in cognitive domains including attention, memory, and semantic processing. Furthermore, the LLFS offspring generation has significantly lower rates of major diseases of aging, including diabetes, chronic pulmonary disease, and peripheral artery disease, and shows significantly more favorable profiles of quantitative traits of health aging such as blood pressure, lipids, functional performance, and cognitive indices compared with the FHS.

The cross-sectional and longitudinal heritabilities for key healthy aging phenotypes are shown in Table 3. For most phenotypes, the cross-sectional heritabilities are roughly equal at both visits, and depending on phenotype, ranging from a low of 0.08 (SPPB physical performance battery at Visit 2) to a high of 0.55 (for BMI at Visit 2). Most cross-sectional heritabilities are in the 0.30–0.40 range. In general, the RCM slope heritabilities are substantially higher than the corresponding OLS slope heritabilities (Table 3). As noted, this is because the growth curve approach properly models errors in measurement at each time point, whereas the more traditional OLS approach ignores potential measurement error and takes measurements at face value, which results in measurement errors accumulating rather than canceling when looking at the differences between time points. The increased error in estimating longitudinal change results in loss of precision and lower heritability for the OLS approach.

## Discussion

LLFS is a unique multicenter, multinational resource for discovering protective alleles and associated biological signatures for Healthy Aging Phenotypes (HAPs) and Exceptional Longevity (EL) (4,5). No other study has LLFS’ size and degree of exceptional familial longevity, as fewer than 1% of Framingham Heart Study (FHS) families would meet the EL selection criteria for LLFS (6). LLFS cohort is comprehensively and broadly and deeply phenotyped on all of the major domains of healthy aging.

### Evidence That LLFS Families Are Healthier than Average for Their Age and Sex

In Figure 1, we demonstrate that the LLFS cohort is far healthier on average than a random sample reference pedigree cohort (Framingham Heart Study) in many key phenotypes of healthy aging. In published findings of the cross-sectional Visit 1 data, LLFS probands and offspring were less likely to have diabetes, chronic pulmonary disease, and peripheral artery disease than the Cardiovascular Health Study (CHS) and FHS cohorts (8). Measures of physical function and cardiovascular risk factors were on average more optimal in LLFS compared with the other groups. High-density lipids were higher, and pulse pressure and triglycerides were lower in LLFS probands and offspring. The perceptual speed task, gait speed, and cognitive function assessed by the Digit Symbol Substitution Test (DSST) were significantly better in LLFS (8). LLFS offspring were shown to be cognitively healthier than spouse controls across a number of different cognitive domains (29,30). Furthermore, Barral et al. (31) showed that the exceptional cognition LLFS families are enriched with healthier metabolic profiles, suggesting that pleiotropic effects of rare alleles may be underlying this phenomenon. Ash et al. (32) demonstrated a lower prevalence of many diseases in LLFS participants using Centers for Medicare and Medicaid (CMS) data compared with non-LLFS Medicare beneficiaries, and Singh et al. (33) demonstrated similar findings when comparing LLFS to Health ABC. In fact, combining all disorders, LLFS participants show increased health-span compared with population controls and comparable to centenarians (5).

### Evidence That Protective Familial Effects in Danish Families Persist Into the Third Generation

Using the Danish Civil Registration System and parish birth records (34), we previously identified 3 638 long-lived families with at least 2 living siblings. We interviewed family members and established the pedigrees of 659 families including the 76 LLFS Danish families. We obtained the socioeconomic status, all hospitalizations, and causes of death for offspring and grandchildren of the long-lived siblings and their 10 age- and sex-matched controls for each offspring and grandchild via the nationwide Danish registers. Our first analyses demonstrated that offspring of Danish long-lived siblings had a low incidence of tobacco-related cancers (35). We then showed that the offspring as well as their spouses had a lower mortality rate than the background population (34). Recently, we focused on the grandchildren generation, who exhibited significantly lower mortality (0.60% in grandchildren vs 0.85% in controls; *p* = .045) and significantly lower risk of hospitalization (hazard ratio = 0.9; 0.85–0.97 95% CI) during childhood (0–15 years) (36). To better understand the mechanisms underlying this familial transmission of exceptional health and survival in 3 generations, we expanded the study, again using the nationwide Danish registers, and assessed the socioeconomic characteristics of these families and all hospitalizations and causes of death since the 1970s. Comparing the long-lived siblings and their 5 379 offspring and 10 398 grandchildren to the background population controls and spouse controls, we found only small economic and educational differences. However, we consistently noted the lower occurrence of early parenthood, divorce, and lower rates of virtually all disease groups in the offspring and grandchildren of long-lived siblings. We noted that the incidence of hospitalization for mental and behavioral disorders was lower by approximately half the expected rate in the offspring (data not shown) and by a quarter in the grandchildren. Controlling for educational achievements only changed the estimates marginally for nearly all disease groups in both generations. Thus, remarkably, the grandchildren generation appears to also exhibit relatively enhanced healthy aging.

### Evidence That LLFS Healthy Aging Phenotypes Are Heritable, Cross-Sectionally and Longitudinally

We demonstrated in Table 3 that key healthy aging phenotypes in the LLFS cohort are heritable, both cross-sectionally and longitudinally. However, as seen in Table 3, the longitudinal heritability is severely attenuated using a simple “difference between values” (OLS Slope) way of calculating individual change. In fact, for change calculated this way, heritabilities are often close to zero. The longitudinal heritability is recovered to nearly the corresponding cross-sectional magnitudes when the longitudinal change is estimated using the Random Coefficient Models (aka Growth Curves), as detailed in the Method section and Supplementary Materials. The reason this approach works better is that it takes into account the fact that each visit data point is measured with (at least some) error. Because calculating change between 2 measurements on the same person amounts to a “distance” between measured values, for such distances, errors of measurement in the individual data point errors tend to accumulate in the distance measure, they do not tend to cancel. So the naive way to calculate “change” is estimated with much more error than each of the 2 individual values themselves, and this large error overwhelms the ability to estimate heritability. In fact, several other studies of longitudinal change in some of these same phenotypes have found much smaller heritabilities using the simple “change in values” way of estimating change (37,38). The individual growth curve approach correctly models these individual measurement errors and reduces the error in the estimated change, which allows heritability to be estimated with greater accuracy.

### Evidence That LLFS Families Are Heterogeneously Protected

Although we have demonstrated above that *on average* the LLFS cohort is healthier than random cohorts of the same age/sex, they are not uniformly so. In particular, the pedigrees that show the most protection in 2 different phenotypic domains are not always the same ones. For instance, of the 539 LLFS families, we identified 18 as Exceptional Memory families (*N* = 405 individuals) that showed significant familial clustering of exceptional verbal episodic memory (10). On the other hand, we found 44 families (*N* = 306 individuals) that showed significant familial clustering for healthy blood pressure (13). Similarly, 42 families were found to demonstrate significant clustering for exceptional grip strength and 37 families with exceptional FEV_1_. There is some, but relatively little, overlap between exceptional families across different phenotypes, suggesting heterogeneous mechanisms of healthy aging (9).

### Evidence That Multiple, Rare, Protective Variants Likely Drive Some HAPs and Longitudinal Trajectories in Selected LLFS Families

Linkage analysis is used to identify broad genomic regions that may harbor a causal variant by examining pedigrees in which correspondences between allele sharing identity-by-descent (IBD) and phenotype similarity, which is recently regaining interest with the focus on rare variation (39). We are beginning to conduct nonparametric linkage analyses of LLFS pedigrees for survival as well as key HAPs. Initial analyses have produced strong linkage peaks, most notably for carotid ultrasonography quantitative measures of atherosclerosis (40). Detailed analyses of these peaks suggest that they are likely driven by rare, lineage-specific variants, running in select pedigrees. Three pieces of evidence support this hypothesis. First, very little of the linkage evidence is accounted for by regressing out all nearby associated Genome Wide Association Scan (GWAS) or 1000 Genomes imputed SNPs even if we use very weak thresholds of significance far below the GW level. The comprehensive density of our 2.5M GWAS panel (imputed to 38M SNPs) suggests that it is unlikely that there are very many “hidden” common variants that could have escaped tagging or imputing by our dense GWAS and still be driving these linkage peaks. On the other hand, it is well known that rare variants are not well tagged by the (mostly) common variants in GWAS chips, nor are extremely rare ones imputed very accurately (41). Second, even though these peaks are highly statistically significant in all families (eg, Logarithm of the ODDs [LOD] = 5.3 for interadventitial diameter [IAD] on 3q131), the majority of evidence seems to be strongly concentrated in a few, select families, as demonstrated by family-specific heterogeneity LOD scores (HLOD) (42). For example, the IAD HLOD = 7.8 in just 23 of the 539 LLFS families with carotid ultrasound phenotypes (the remaining families have near-zero or negative HLODs, suggesting that there is no linkage for them). Given the phenotypic heterogeneity we previously found in selected pedigrees (above), and the general locus heterogeneity of most complex traits, our linkage heterogeneity would not be surprising. Finally, we can demonstrate through simulation experiments, that clusters of rare lineage-specific causal variants segregating in pedigrees can collectively produce a strong linkage peak, but the signal is completely missed by standard GWAS analysis (Supplementary Appendix 2). Taken together, these findings suggest that our linkage peaks are driven by relatively few, rare nearly lineage-specific loci clustered in the same gene or regulatory region, segregating in a few families with strong linkage evidence, while the remaining families have zero or negative LOD score evidence because they do not carry any of the driving rare variants (generally, families with pedigree-specific LOD scores > 0.2 are the driving linkage families). Furthermore, the fact that the LLFS cohort is on average healthier than random cohorts for many phenotypes, and that the phenotypic heterogeneity we see in LLFS is pedigree-specific and is manifested by exceptionally favorable HAPs in selected pedigrees, suggests that if there are rare variants driving the linkage peaks, they are likely protective variants. This makes LLFS a potentially powerful platform for rare protective variant discovery.

In summary, LLFS is uniquely positioned to discover genetic and other factors related to longevity and exceptional “health span” and the mechanisms that contribute to them. The role of rare genetic variants in longevity and exceptional health span has not been thoroughly examined. To date, replicated associations of common variants with longevity have been few and their effect sizes have been modest. It is now clear that rare variants are numerous and many are of recent origin (43–45) and that family studies show particular promise in discovering these (46). LLFS has developed a strategy to utilize its family genetic data efficiently and intensively to identify both coding and regulatory variants that contribute to EL and/or HAPs.

### Future Directions

LLFS has recently obtained funding to continue annual follow-up and conduct a third longitudinal in-person visit of all participants. Visit 3 will repeat all Visit 2 measurements and protocols and add a few new ones, including formal dementia diagnosis and dietary assessment. In Visit 3, we will also recruit members from the grandchildren generation in selected pedigrees where we have strong LOD score linkage evidence for key HAPs. Whole-genome sequencing will be done on all pedigrees and participants, and metabolomics (via mass spectrometry) will be done longitudinally on all participant samples, including stored blood from Visits 1 and 2, as well as new blood collected for Visit 3. Other OMICs will also be done longitudinally on samples from Visits 1, 2, and 3, in selected pedigrees showing strong evidence for linkage for key HAPs. These include transcriptomics (RNA-seq), epigenomics (Whole-Genome Bisulfate Sequencing), and proteomics (either SOMAScan or mass spectrometry, depending on the results of harmonization efforts across sister studies of longevity and healthy aging). These will allow us to better identify the rare causal variants that appear to be running in selected LLFS pedigrees and, more importantly, identify the genes of action and the biological mechanisms behind the protection. Finally, an important advantage afforded by the LLFS family design is that we have obtained a sample of younger participants (in the offspring, and soon, the grandchildren) that appear to be enriched for protection in many dimensions. They may (or may not) ultimately manifest extreme longevity, but they are an important sample for longitudinal tracking, as they may provide a mechanism for prospective discovery of protective biomarkers and signatures, and for understanding the interplay of exposures, lifestyle, and biology in the etiology of healthy aging and longevity.

## Funding

This work was supported by National Institute on Aging (grants U01AG023746, U01AG023712, U01AG023749, U01AG023755, U01AG023744, and U19 AG063893).

## Author Contributions

M.K.W.: data management, statistical analysis, draft manuscript. S.J.L.: data management/quality control, statistical analyses, produced figures. P.S.: ascertainment criteria, statistical analyses, edit manuscript. T.T.P.: collected clinical data, edit manuscript. J.L.: collected clinical data, edit manuscript. K.C.: collected clinical data, edit manuscript. M.A.P.: conceived project, draft manuscript, edit manuscript. A.K.: edited manuscript. A.N.: collected clinical data, edit manuscript. J.M.Z.: collected clinical data, edit manuscript.

## Conflict of Interest

None declared.

## Supplementary Material

glab333_suppl_Supplementary_Materials_S1
