# Supplementary material for: NIA Long Life Family Study: Objectives, Design, and Heritability of Cross-Sectional and Longitudinal Phenotypes
Source: J Gerontol A Biol Sci Med Sci. 2021 Nov 5;77(4):717–27. doi: 10.1093/gerona/glab333 (PMC8974329; doi:10.1093/gerona/glab333)
Supplement: glab333_suppl_Supplementary_Materials_S1 [file glab333_suppl_supplementary_materials_s1.pdf]

Appendix 1: Random Coefficient Model (RCM a.k.a. Growth Curves)

For the ith person, $i=1, 2, ..., N$, measured at T_i personal time points $t=1, 2, . . ., T_i$ (note, the time points are personal in that each person may have a different number of time points, they may be at differing intervals between them, and they may occur at completely different times from those of other participants). Then $T = \sum_{i=1}^{N} T_i$ is the total number of phenotypic measurements across all subjects and time points. Let $Y_{i,t}$ and $Age_{i,t}$ denote the phenotypes and the corresponding Ages of the ith person at their personal time of measurement t. We call the first time point for each person their "baseline." Denote the time after baseline as $T_{i,t} = (Age_{i,t} - Age_{i,1})$. Let $X_{i,t}$ denote a set of k relevant covariates for the ith person measured at time t, that may be used to predict the phenotype (such as Baseline Age=$Age_{i,1}$, sex, race, exposures, etc. as well as any higher order interactions between these). A linear Growth Curve is a mixed linear model $Y = X\delta + Z\gamma$ in which we have a set of k fixed effect covariates $X_{i,t}$ as well as two random effects, one for random intercepts and one for time after baseline, $T_{i,t}$. This model assumes that every subject, i, has their own personal linear trajectory, with a personal intercept and slope, α_i and β_i , respectively), with independent errors, $\varepsilon_{i,t}$. For unrelated (independent) subjects, for each i, t

$$Y_{i,t} = X_{i,t}\, \underline{\delta} + [\, \alpha_i + \beta_i\, T_{i,t}] + \varepsilon_{i,t}$$

The random effects part of the model makes the additional assumption that

$\begin{pmatrix}\alpha_i\\ \beta_i\end{pmatrix}$ is independent of $\begin{pmatrix}\alpha_j\\ \beta_j\end{pmatrix}$ for $i \neq j$ and within each subject i :

$$\begin{pmatrix}\alpha_i\\ \beta_i\end{pmatrix} \sim N\left[\begin{pmatrix}\alpha\\ \beta\end{pmatrix}, \begin{pmatrix}\sigma_\alpha^2 & \rho\sigma_\alpha\sigma_\beta\\ & \sigma_\beta^2\end{pmatrix}\right]$$

where α and β are the fixed effect intercept and slope (over time after baseline), respectively, σ_α^2 and σ_β^2 are the variances of the personal (random) intercepts and slopes, respectively, and ρ is

the correlation between personal (random) slopes and intercepts for the same subject. The model is fit using maximum likelihood.

For longitudinal pedigree data, such as LLFS, the kinship matrix can be added as an additional random effect to account for the non-independence within pedigrees. Alternatively, the family bootstrap method[1] can be used on the above mixed model for independent subjects to achieve the same goal, with asymptotically equivalent results.

Appendix 2: Clusters of Multiple Rare variants can produce Linkage without GWAS association

We hypothesize that multiple rare causal variants for complex traits may cluster in a single gene (or region), much as they often do for monogenic traits (e.g. BRCA1, CFTR, DMD, etc.) If this happens, the multiple rare variants may be individually too small in effect size or too low in LD with GWAS SNPs to be found in a GWAS screen since common variants are poor tags for rare ones[2], but they may combine sufficiently strongly to produce a strong linkage peak. This would explain our and many others' findings of discordance between linkage and GWAS associations. To verify whether this is possible, we simulated data using software we developed for the Genetic Analysis Workshop 16[3], using real GWAS data, and real pedigree structures from the FHS, but simulated complex trait phenotypes from multiple causal variants that we selected throughout the genome. We simulated traits with heritabilities in the 40-60% range, caused by hundreds to thousands of polygenic SNPs throughout the genome, each explaining fractions of a percent of the variance. We specifically selected clusters of 9 causative mutations to be in a single gene, and examined the net effect on linkage as well as to GWAS analysis. In **Appendix 2 Figure 1** we show the linkage and GWAS results from one such simulation scenario, in which, for each of the

9 largest pedigrees, we chose a different SNP to be causative for that lineage. To make the 9 causative variants “rare”, we recoded those 9 SNPs to be homozygous to the common allele in all other families. We then removed the 9 causative SNPs from the GWAS analysis since variants on GWAS chips tend to be tags. We simulated a trait with locus-specific heritabilities of 2% at each of these 9 variants, for 18% total explained by this super-locus. The 5 solid lines show the linkage analyses for the first 5 replicates (typical of the 100 replicates). A partial GWAS Manhattan plot association of the non-causative SNPs from the 1st replicate (-log P > 2 only) is overlaid. In 100 replicates, the median LOD score in the region was 2.43 (s.d. 1.04, max 6.86, min 0.511), while the –log p value for GWAS markers within 10MB of the true causal variants was < 6, which is well below the 10^{-8} GW significance level. This signal is just detectable by linkage, but well below the GWAS threshold. Most importantly, the very rare family specific causal mutations themselves were significant between $P=10^{-10}$ and $P=10^{-40}$ using our proposed family mixed model association test. Thus, when we actually sequence such rare variants, we have excellent power to detect association. Only because we used family data with multiple members segregating the rare mutation, were we able to detect them through linkage in the first place. These results demonstrate the importance of family data and suggest that family-based association tests will be very useful in identifying rare mutations in sequence data. They also show that if multiple rare variants cluster in the genome they may produce a strong linkage peak but be missed by GWAS.

Appendix 2 Figure 1. Simulation of Linkage Peak caused by

Mulitple Rare Variant Loci Clustered in a Single Gene Region

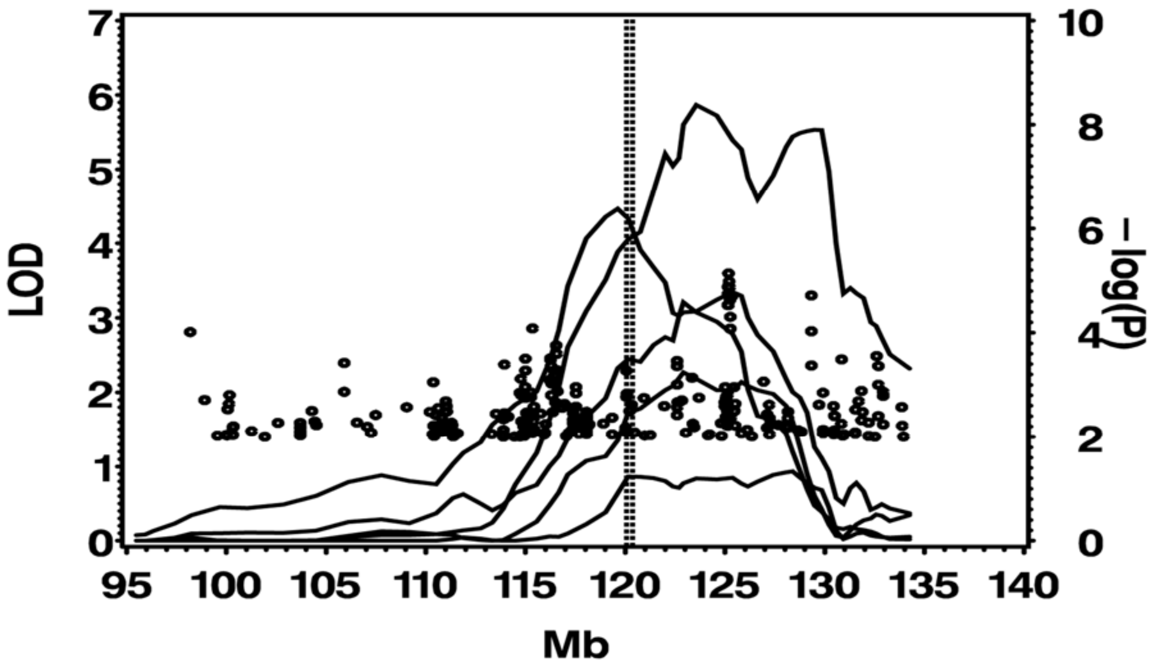

Legend:

Mb=Mega-base position on chromosome

Shaded area at 120 Mb = Cluster of rare causal variants for the simulated phenotype

left y axis LOD=LOD score (significance of linkage). LOD scores at each bp location are given by the solid connected lines (each line represents a different simulation using the same cluster of rare variants, and show the variability in linkage evidence possible in producing phenotypes). LOD> 3 is traditionally genome-wide linkage significance.

right y-axis -log(p)=- minus log base 10 of the association p-value in GWAS. GWAS results are shown as Manhattan plot solid dots (unimpressive –log10(p) values < 2 are suppressed to avoid overcrowding. A –log10(p)>8 is traditionally genome-wide association significance

REFERENCES for Appendicies

1. Borecki IB and Province MA. Genetic and genomic discovery using family studies. *Circulation*. 2008;118:1057-63.

2. McCarthy MI and Hirschhorn JN. Genome-wide association studies: past, present and future. *Hum Mol Genet*. 2008;17:R100-1.

3. Kraja AT, Culverhouse R, Daw EW, Wu J, Van Brunt A, Province MA and Borecki IB. The Genetic Analysis Workshop 16 Problem 3: simulation of heritable longitudinal cardiovascular phenotypes based on actual genome-wide single-nucleotide polymorphisms in the Framingham Heart Study. *BMC Proc*. 2009;3 Suppl 7:S4.
